# Effectiveness of Autologous Stem Cell Therapy for the Treatment of Lower Extremity Ulcers: A Systematic Review and Meta-Analysis

**DOI:** 10.1097/MD.0000000000002716

**Published:** 2016-03-18

**Authors:** Xupin Jiang, Hengshu Zhang, Miao Teng

**Affiliations:** From the Institute of Burn Research (XJ), State Key Laboratory of Trauma, Burns and Combined Injury, Southwest Hospital, The Third Military Medical University; and Department of Burn and Plastic Surgery (HZ, MT), The First Affiliated Hospital of Chongqing Medical University, Chongqing, China.

## Abstract

Primary studies in animal models and humans have suggested the therapeutic potential of autologous stem cell for treating chronic lower extremity ulcers. However, the results of pilot randomized controlled trials (RCTs) in humans have been inconsistent.

A meta-analysis of RCTs was performed to evaluate the role of autologous stem cell-based therapy for lower extremity ulcers.

Studies were identified during a systematic search of Medline, Embase, Cochrane's library, and references cited in related reviews and studies.

Studies were included if they were RCTs published in English, recruited patients with lower extremity ulcers who were assigned to either a group for the topical therapy with autologous stem cells, and reported data regarding the healing of the ulcers.

Relative risks (RRs) for healing rate and standardized mean differences (SMDs) for the changes in the mean sizes of ulcers were evaluated with a random-effects model.

Overall, autologous stem cell-based therapy was associated with better healing of lower extremity ulcers (12 comparisons, 290 patients, RR for partial healing = 3.07, 95% confidence interval [CI] = 1.14–8.24, *P* = 0.03; RR for complete healing = 2.26, 95% CI = 1.48–3.16, *P* < 0.001) with little heterogeneity (*I*^2^ = 0%). Moreover, autologous stem cell-based therapy was associated with a greater reduction in mean ulcer size (SMD = −0.63, 95% CI = −1.03 to −0.22, *P* = 0.002). Subgroup analyses indicated that stem cells from peripheral blood and bone marrow seemed to exert similar beneficial effects on the healing of ulcers. Stem cell therapy was not associated with any increased risks for adverse events.

The optimized sources, amounts, and delivery methods of stem cell -based therapy for patients with chronic lower extremity ulcers need to be determined, and the long-term effects of stem cell-based therapy on clinical outcomes need further exploration.

Autologous stem cell-based therapy is effective and safe for improving the healing of chronic lower extremity ulcers and large-scale RCTs are needed to confirm our findings.

## INTRODUCTION

Diabetes mellitus (DM) and peripheral arterial disease (PAD) have been recognized as 2 of the most important causes of lower extremity ulcers.^1,2^ For patients with DM, a diabetic foot ulcer (DFU) is a common and serious complication. Previous studies indicated that ∼15% to 25% of diabetes patients will develop a DFU during their lifetime, thereby exposing them to the increased risks of amputation and death.^3,4^ For patients with advanced PAD, rest pain and ulcers occur in the ischemic lower extremity, which is clinically defined as critical limb ischemia (CLI).^5^ Similarly, ∼30% patients with CLI will undergo amputations of the affected limbs.^6^ Moreover, CLI patients also have a poor prognosis, because they have an increased risk of cardiovascular-related mortality.^2,6^ Despite treatment of the original diseases, healing of lower extremity ulcers in DM and PAD patients is difficult to achieve.^7^ The current standard of care for lower extremity ulcers includes debridement, local wound care, prophylaxis, and treatment of infection, as well as off-loading the pressure.^1,8,9^ Despite improvements in the treatment strategies for wounds or skin ulcers during the past decades, an effective treatment for promoting healing of a wound, particularly those caused by DM and CLI, is generally lacking. Moreover, the delayed healing of a lower extremity ulcer may lead to complications of infection, necrosis, or even sepsis, which may lead to amputation or death.^10,11^ Therefore, novel treatment strategies that can accelerate the healing of skin wounds are needed.

Autologous stem cell-based therapy has been proposed as a promising strategy for the treatment of topical lower extremity ulcers by pilot studies in animal models and humans,^9,12–14^ because the stem cells may influence many pathophysiologic processes involved in the healing of ulcers, including stimulating the activities of tissue repair cells, increasing the synthesis of extracellular matrix (ECM) and release of growth factors, and promoting angiogenesis in the ischemic tissue.^12,13^ Indeed, some small-scale clinical trials published recently have investigated the potential efficacy and safety of applying multipotent adult stem cells to promote the healing of lower extremity ulcers. However, the results have been inconsistent, and the interpretation of the results may be biased by the limited statistical power of the studies.^15–24^ Moreover, whether stem cells derived from different sources may confer different efficacies in this condition also needs to be confirmed.^25^ Therefore, in this study, we performed a systematic review and meta-analysis of the published randomized controlled trials (RCTs) to evaluate the role of autologous stem cell-based therapy in the treatment of lower extremity ulcers.

## METHODS

### Ethics Statement

This study was approved by the First Affiliated Hospital of Chongqing Medical University Ethics Committee. This study does not involve patients, so ethical approval was not required.

The primary objective of the present study was to comprehensively investigate the therapeutic efficacy and safety of topical application of autologous stem cells in patients with lower extremity ulcers. This meta-analysis was performed according to the PRISMA (Preferred Reporting Items for Systematic Reviews and Meta-Analyses) statement^26^ and the Cochrane Handbook guidelines.^27^

### Literature Search

We systematically searched the Pubmed, Embase, and Cochrane Library (Cochrane Center Register of Controlled Trials) databases for related studies, using the terms “stem cell,” “progenitor cell,” “bone marrow,” “mononuclear cell” paired with “foot,” “lower extremity” and “wound,” or “ulcer.” The searching was limited in studies of humans. The final search was performed on February 28, 2015. We also manually searched the references of the original and review articles for possible related studies.

### Study Selection

Studies were included if they met all of the following criteria: (1) published as a full-length article or abstract in English; (2) reported as an RCT; (3) recruited patients with lower extremity ulcers who were assigned to either a group for the topical therapy with autologous stem cells (derived either from bone marrow or from peripheral blood) or a control group (with no treatment or placebo); and (4) reported outcomes regarding the healing of the ulcers (including the incidences of partial or complete healing of the ulcers, or the changes in ulcer size in both groups), or the relevant data could be estimated. Safety outcomes reflected by adverse events related to the autologous stem cell-based therapy were also extracted. Reviews, case reports, and other studies not designed as RCTs were not included for the current analysis.

### Data Extraction and Quality Assessment

Two of the authors (XJ and HZ) independently conducted the literature search, data extraction, and quality assessment process according to the predefined inclusion criteria. Discrepancies among the authors were solved by discussion with the third author (MT). Data regarding study design characteristics (blind or open-label, placebo-controlled or not), countries of the studies, characteristics of the included patients, numbers of participants, underlying causes of lower extremity ulcers, baseline sizes of ulcers, details of autologous stem cell-based therapy (sources of stem cells, amounts of cells applied, and delivery strategies), follow-up duration, as well as incidences of adverse events were extracted. We applied the 7 domains of the Cochrane risk of bias tool for the quality evaluation of the included studies. This quality evaluating strategy included criteria concerning aspects of sequence generation, allocation concealment, blinding of participants and personnel, blinding of outcome assessors, incomplete outcome data, selective outcome reporting, and other potential threats to validity.^27,28^

### Statistical Analyses

We used risk ratios (RRs) with 95% confidence intervals (CIs) for the analyses of dichotomous data, whereas the continuous data were presented as standardized mean difference (SMD) with 95% CIs. We applied Cochrane's *Q* test for the evaluation of the heterogeneity among the studies, and a *P* < 0.10 was set as significant heterogeneity among the included studies. Moreover, the *I*^2^ statistic,^29^ which was considered to estimate the percentage of heterogeneity derived from inter-study heterogeneity instead of chance, was also determined. We defined *I*^2^ > 50% as an indicator of significant heterogeneity among the trials.^30^ We used random-effects models to estimate the pooled results to minimize the influence of potential clinical heterogeneity among the studies. Subgroup analyses were performed to evaluate the influence of certain study characteristics on the outcomes. Sensitivity analyses^27^ were also performed to evaluate the robustness of the polled results, by subsequently removing individual study. Potential publication bias was assessed with Egger's regression asymmetry test and visual evaluation of funnel plots.^31^ Subsequently, we also performed the nonparametric “trim and fill” analysis^27,30^ to further assess the possible influence of publication bias in our meta-analysis by estimation and incorporation of the hypothetical missed studies with negative results. Statistical significance was defined as a 2-tailed *P* < 0.05. We used RevMan software (Version 5.1; Cochrane, Oxford, UK) and Stata software (Version 12.0; Stata, College Station, TX) for the statistical analysis.

## RESULTS

### Search Results

**FIGURE 1:**
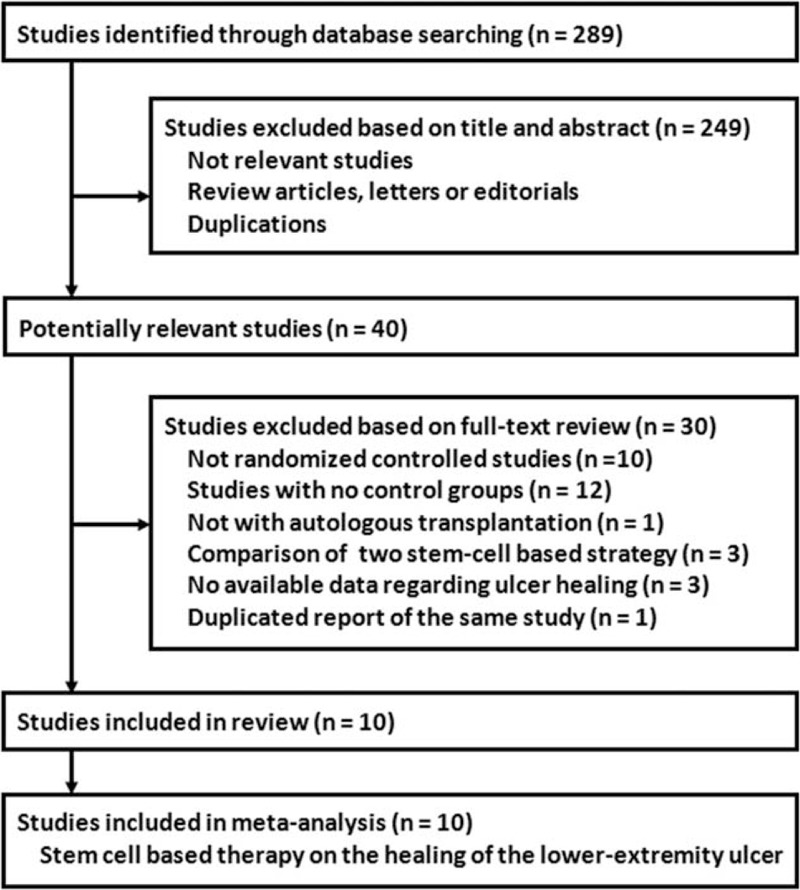
Flow diagram of the study selection procedure.

The literature screening process is outlined in Figure 1. Briefly, a total of 289 studies were identified in the initial database search, and 249 were excluded mainly because they were not relevant to the objective of the study. Of the 40 potentially relevant studies, 10 studies^15–24^ met the inclusion criteria for the current meta-analysis. Thirty studies were further excluded because 10 were not RCTs, 10 did not include control groups, 1 did not use autologous stem cells, 3 compared 2 cell-based strategies, 3 did not report relevant data, and 1 was a duplication of another study.

### Study Characteristics

The general characteristics of the included studies are listed in Table 1 . One study (Dash 2009)^16^ reported data regarding 2 subgroups of patients according to different causes of lower extremity ulcers; therefore, 2 comparisons were derived. Moreover, another 3 studies reported 2 interventional arms according to different types (Lu 2011 and Kirana 2012)^18,21^ or doses (Losordo 2012)^22^ of stem cells used, and similarly, 2 comparisons were derived for each. The sample size of the control group was therefore equally distributed to the 2 study arms to overcome a unit of analysis error as recommended in Cochrane's Handbook.^27^ Overall, 14 comparisons, with 311 patients with lower extremity ulcers, were included in the meta-analysis. The studies were performed in China,^15,18,24^ the USA,^19,22^ Germany,^20,21^ India,^16,17^ and Egypt.^23^ The mean age of the included patients varied from 40 to 71 years, with proportions of males ranging from 37% to 85%. The cause of the lower extremity ulcers was CLI for most of the studies,^15,18–24^ but patients with Buerger's disease,^16^ DFU,^16,17^ as well as traumatic ulcers^17^ were also included. As for the autologous stem cells applied, 4 comparisons used granulocyte colony-stimulating factor (G-CSF)-mobilized peripheral blood mononuclear cells (PBMNCs),^15,22,23^ 3 used bone marrow-derived mesenchymal stem cells (BMMSCs),^16,18^ 4 used bone marrow-derived mononuclear cells (BMMNCs),^18,20,21,24^ 2 used bone marrow-enriched tissue repair cells (BMTRCs),^19,21^ and 1 used bone marrow-derived stem cells.^17^ Both the intra-arterial and the intramuscular routine were applied for topical stem cell-based therapy in the studies. The follow-up duration varied from 6 to 48 months. Only 1 study^19^ reported possible adverse events related to autologous stem cell-based therapy (including pain in extremity, nausea, gangrene, cellulitis, diarrhea, and localized infection), and the incidence of such adverse events was not different between the stem cell-based therapy and placebo groups.

**TABLE 1:**
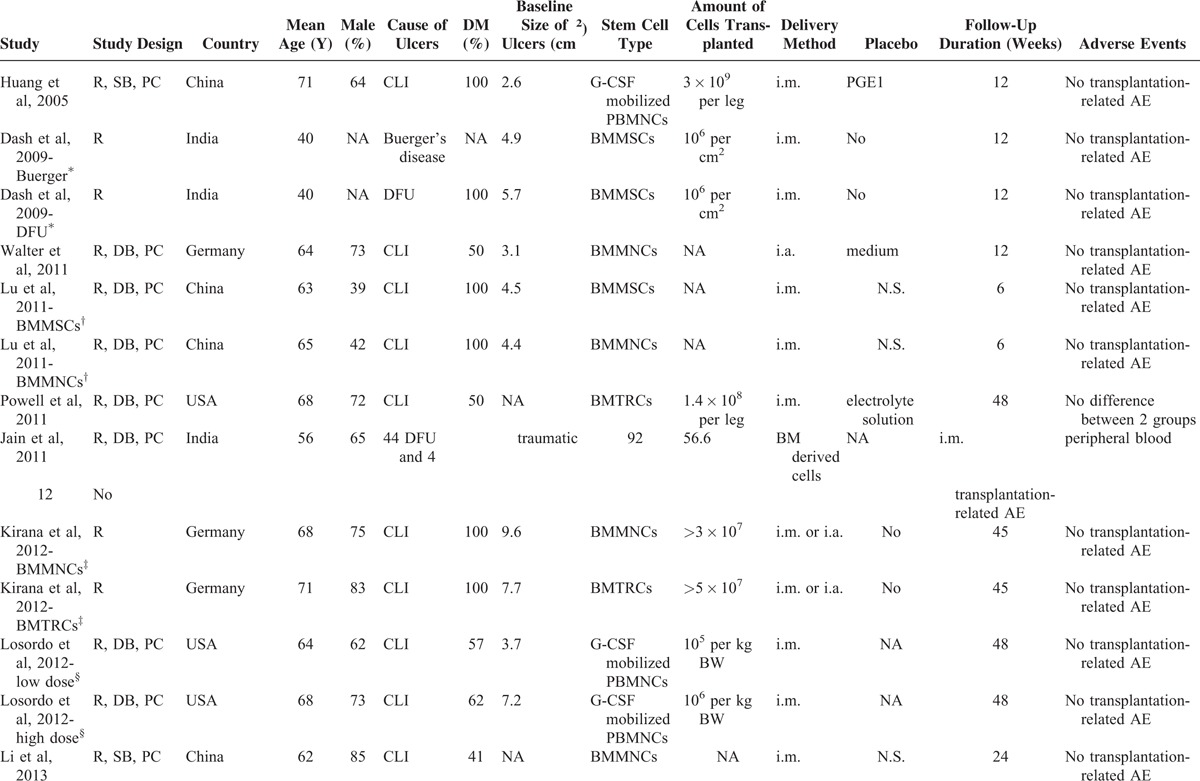
Characteristics of the Included Studies

**TABLE 1 (Continued):**
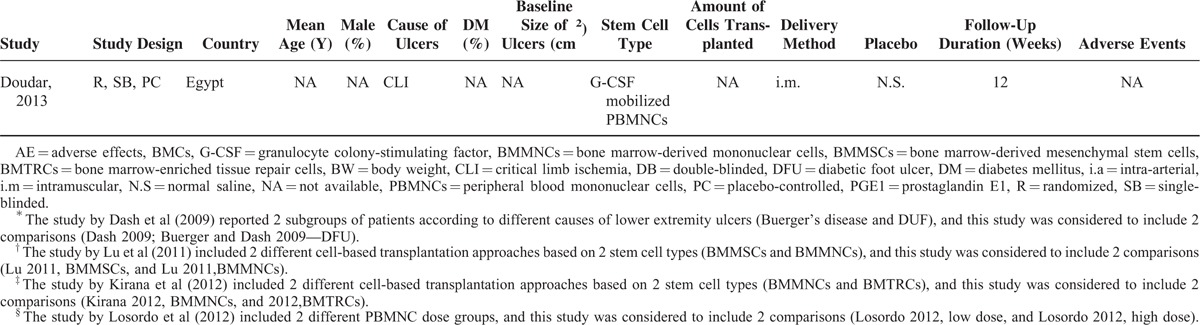
Characteristics of the Included Studies

### Quality Assessment

The risks of biases of the included studies as evaluated by the Cochrane assessment tool are shown in Table 2. Five of the included RCTs were double-blinded, placebo-controlled studies.^17–20,22^ Only 2 studies reported methods of random sequence generation and the details of allocation concealment.^16,17^ Details of withdrawals and dropouts were reported in all studies. We were uncertain of the potential bias of selective outcome reporting for the included studies, because none of them published a protocol before the performance of the studies. However, we believed that the influence of potential bias from selective outcome reporting on the overall effect of stem cell-based therapy was insignificant given that we focused on the efficacy of the therapy for healing of the ulcers, which was reported in all of the included studies.

**TABLE 2:**
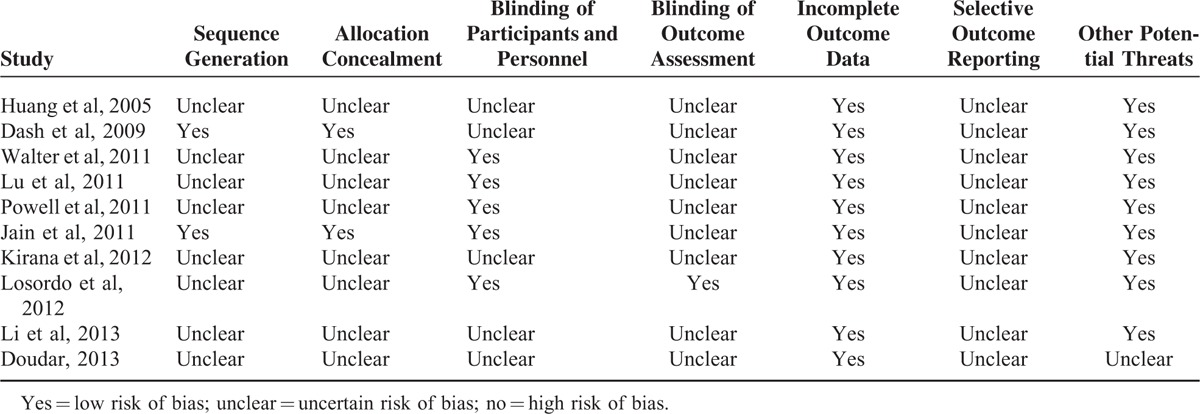
Cochrane Risk of Bias Assessment

### Effects of Autologous Stem Cell Therapy on the Healing of Lower Extremity Ulcers

**FIGURE 2:**
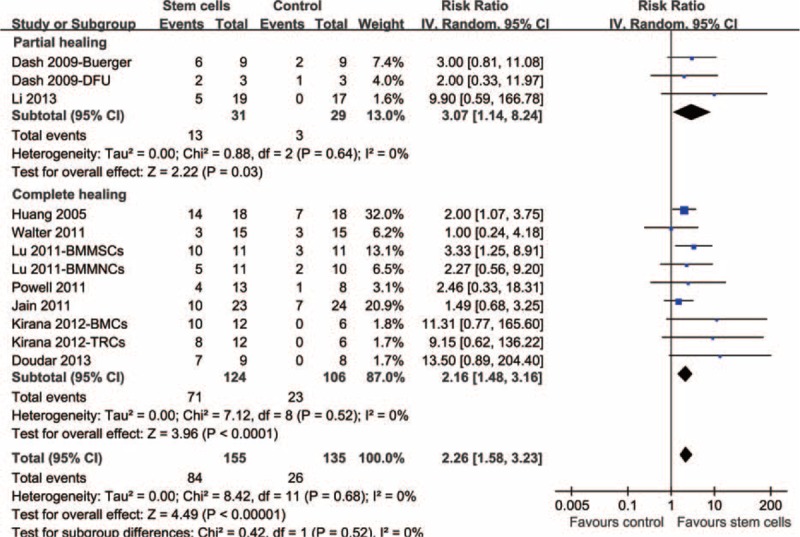
Forest plot from meta-analysis of risk ratio (RR) of partial or complete healing of lower extremity ulcers in patients randomized to the autologous stem cell-based therapy or control groups. RR = risk ratio.

Twelve comparisons,^15–21,23,24^ with 155 patients assigned to the stem cell-based therapy group and 135 to the control group, investigated the effects of autologous stem cell therapy on the healing of lower extremity ulcers. Overall, the results of the meta-analysis with a random-effects model indicated that autologous stem cell-based therapy was associated with better healing of lower extremity ulcers (12 comparisons, 290 patients, RR = 2.26, 95% CI = 1.58–3.23, *P* < 0.001; Figure 2) as compared to that observed in the controls, and no significant heterogeneity was found among the included studies (Cochrane's Q test *P* = 0.68, *I*^2^ = 0%). Stratified by outcomes, autologous stem cell-based therapy was associated with improved rates of both partial healing (3 comparisons,^16,24^ 60 patients, RR = 3.07, 95% CI = 1.14–8.24, *P* = 0.03) and complete healing (9 comparisons,^15,17–21,23^ 230 patients, RR = 2.26, 95% CI = 1.48–3.16, *P* < 0.001). No significant heterogeneity was detected for either of these subgroups (*I*^2^ = 0% for both subgroups; Figure 2).

**FIGURE 3:**
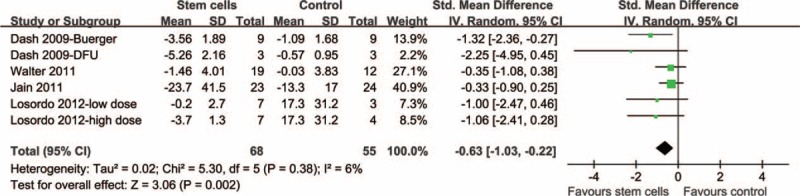
Forest plot from meta-analysis of standardized mean difference (SMD) of lower extremity ulcer size in patients randomized to the autologous stem cell-based therapy or control groups. SMD = standardized mean difference.

Six comparisons^16,17,20,22^ including 123 patients investigated the effects of autologous stem cell therapy on the changes in the mean size of lower extremity ulcers. The pooled results with a random-effects model showed that autologous stem cell-based therapy was associated with a greater reduction in mean ulcer size as compared with that observed in the controls (SMD = −0.63, 95% CI −1.03 to −0.22, *P* = 0.002; Figure 3) with little heterogeneity among the studies (Cochrane's Q test *P* = 0.38, *I*^2^ = 6%).

### Subgroup and Sensitivity Analyses

We tried to determine whether autologous stem cells from different sources were all associated with better complete healing rates of lower extremity ulcers. The results of the subgroup analyses showed that therapy with autologous G-CSF-mobilized PBNMCs (2 comparisons,^15,23^ RR = 2.20, 95% CI = 1.97–4.07, *P* = 0.01) and with bone marrow-derived stem cells (7 comparisons,^17–21^ RR = 2.13, 95% CI = 1.31–3.47, *P* = 0.002) were associated with better complete healing rates of the lower extremity ulcers. Moreover, the effects of stem cells from the peripheral blood and bone marrow on the healing of lower extremity ulcers seemed to be similar (*P* for subgroup interaction = 0.66). As for the patients with lower extremity ulcers only caused by CLI, stem cell-based therapy was associated with an improved healing rate (RR = 2.51, 95% CI = 1.63–3.87, *P* < 0.001) and a greater reduction in the mean ulcer size (SMD = −0.59, 95% CI −1.18 to −0.01, *P* = 0.04) compared with those observed in control groups. The results of sensitivity analyses conducted by omitting 1 study a time showed that none of the single studies seemed to affect the overall results of the effects of autologous stem cell-based therapy on the healing rate or the change in the size of lower extremity ulcers (data not shown).

### Publication Bias

**FIGURE 4:**
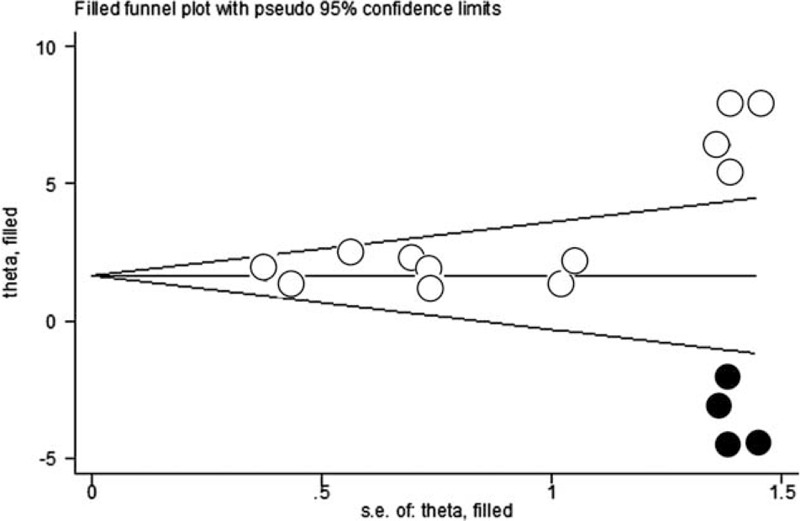
Funnel plot with “trim and fill” for meta-analysis of risk ratio (RR) for partial or complete healing of lower extremity ulcers in patients randomized to the autologous stem cell-based therapy or control groups. The empty dots present the identified studies included in the meta-analysis, and the black dots represent the estimated missing studies after adjustment for publication bias. RR = risk ratio.

The funnel plot for the effects of autologous stem cell therapy on the healing of lower extremity ulcers was asymmetrical on visual inspection (Figure 4), suggesting that potential publication bias could be detected among the included studies. Consistently, the results of Egger's significance tests also indicated the existence of possible publication bias (Egger's regression test *P* = 0.039). Subsequently, we performed a “trim-and-fill” analysis, which imputes 4 hypothetically negative unpublished studies in order to generate a symmetrical funnel plot. The pooled analysis after including the hypothetical studies still showed a statistically improved healing rate for patients who received autologous stem cell therapy as compared with the controls (16 comparisons, RR = 1.82, 95% CI = 1.06–3.12, *P* = 0.031). The publication bias among studies of the effects of autologous stem cell therapy on the changes in mean lower extremity ulcer size was difficult to estimate because only 6 comparisons were included.

## DISCUSSION

It is understood that the healing of the dermal wounds is a complex process that involves many cellular and molecular pathophysiologic events, including the activation and migration of keratinocytes and fibroblasts, synthesis of ECM, production and release of growth factors, regulation and control of inflammation, as well as stimulation of angiogenesis and neovascularization.^32,33^ Previous studies have indicated that the optimal and effective therapy for treating a dermal ulcer may require the coordinated regulation of all of the above key events.^9,13^ Stem cells, as a multipotent cell type that is capable of both self-renewal and multilineage differentiation, have been proposed as a novel treatment strategy for diseases related to tissue regeneration and repair.^12,14^ Indeed, early studies in animal models and observational case series in humans indicated a favorable effect of autologous stem cell-based therapy for the healing of lower extremity ulcers caused by CLI or DM. However, subsequent pilot trials in humans provided inconsistent results, possibly due to the fact that these studies were generally of small scale, and the lack of the statistical power may affect the interpretation of the results.^13^

To overcome the above problem, in this study, we aimed to perform a meta-analysis by analyzing the combined results of available RCTs. We found that autologous stem cell-based therapy was associated with improved healing of lower extremity ulcers, as indicated by greater partial and complete healing rates, as well as a greater reduction in ulcer size, without any increased risk for procedure-related adverse events. Moreover, G-CSF-mobilized PBMNCs and bone marrow-derived stem cells exerted similar beneficial effects on the healing of the ulcers. However, significant publication bias existed, indicating the potential absence of studies with negative results from the literature. The subsequent “trim and fill” analysis after incorporating the imputed studies still favored a beneficial effect of autologous stem cell for treatment of lower extremity ulcers. These results suggested that autologous stem cell-based therapy is a promising and safe treatment strategy for the improved healing of lower extremity ulcers. The results of subgroup analyses and sensitivity analyses further confirmed the robustness of the results. This study, to the best of our knowledge, is the first meta-analysis to provide further evidence that autologous stem cell-based therapy is an effective treatment strategy for improved healing of lower extremity ulcers caused by CLI and DM. Moreover, our results showed that autologous stem cell-based therapy is not associated with an increased risk of procedure-related adverse events during an up to 4-year follow-up duration, suggesting that this novel treatment strategy also seems safe. Obviously, translational studies with a larger sample size and longer follow-up period are warranted.

Despite the significant strengths of the current meta-analysis, some potential limitations should be considered when interpreting the results. First, although our study confirmed the benefits of autologous stem cell-based therapy for the healing of lower extremity ulcers, the optimized procedure characteristics and protocols were not determined in the present study due to the significant heterogeneity among the procedures used in the included studies. Therefore, further studies are needed to determine the optimized sources of stem cells, the proper amount of cells to be delivered, as well as the effective delivery route of the cells. Although our preliminary subgroup analyses indicated that G-CSF-mobilized PBMNCs and bone marrow-derived stem cells exerted similar beneficial effects on the healing of the ulcers, we cannot determine the relative effects of the 2 sources of stem cells because no studies comparing the 2 approaches directly were included in our meta-analyses.^34,35^ Second, our meta-analysis included patients with lower extremity ulcers of different causes and severities, and whether autologous stem cell exerts similar beneficial effects in these clinical conditions needs to be further investigated. Indeed, the etiologies and pathophysiological process of skin ulcers caused by CLI and DM are somewhat different. Whether autologous stem cell confers the same therapeutic benefits deserve further investigation. Third, we focused on the healing of the lower extremity ulcers as a primary outcome in our meta-analysis. Whether a favorable effect of autologous stem cell-based therapy on ulcer healing confers benefits on long-term clinical outcomes, such as reduced risks of amputation or death, needs to be investigated. Moreover, whether autologous stem cell-based therapy confers other benefits, such as improvement in blood supply to the limb in CLI and relief of diabetic neuropathy in DFU, deserves further studies.^36,37^ Fourth, we reported that stem cell-based therapy was not associated with increased risks for adverse events within the follow-up duration up to 4 years. The long-term safety of autologous stem cell-based therapy, particularly with respect to the risk of incident neoplasm, needs to be researched carefully.^38^ Finally, significant publication bias was detected in our study. Although the “trim and fill” analysis including the hypothesized negative trial did not change the overall beneficial effects of autologous stem cell-based therapy on the healing of lower extremity ulcers, further large-scale high-quality RCTs are needed to confirm our results.

In conclusion, the results of our meta-analysis indicate that autologous stem cell-based therapy is effective and safe for improving the healing of lower extremity ulcer caused by ischemia or diabetes.
